# Supplementary material for: Impacts of detritivore diversity loss on instream decomposition are greatest in the tropics
Source: Nat Commun. 2021 Jun 17;12:3700. doi: 10.1038/s41467-021-23930-2 (PMC8211652; doi:10.1038/s41467-021-23930-2)
Supplement: Supplementary file 1 — Supplementary Information [file 41467_2021_23930_MOESM1_ESM.pdf]

Impacts of detritivore diversity loss on instream decomposition are greatest in the tropics

by Luz Boyero et al.

Supplementary Information

Supplementary Tables

Supplementary Table 1. Summary of model selection procedure for the set of models used to examine the influence of detritivore diversity (D), abundance (A), biomass (B) and mean body size (S), latitude (L), and the interactions between the detritivore variables and latitude, on total or detritvore-mediated decomposition, based on Akaike weights (w_i). Only the 10 best models (out of > 500 fitted models) are shown, including the factors and interactions included in each model (+), the degrees of freedom (df), delta AICc (Δ_i; difference in AICc value relative to the best model) and Akaike weights (w_i; probability that a model is the best among the whole set of models). Models are ordered from the best to the poorest fit based on w_i.

Model	Factors and interactions included in the model									df	AICc	Δ_i	w_i
#	D	A	B	S	L	DxL	AxL	BxL	SxL				
**Total decomposition**													
1	+	+	+	+		+	+	+		34	-2530.1	0.00	0.245
2	+	+	+	+	+	+	+	+		36	-2529.7	0.43	0.198
3	+	+			+	+	+	+		33	-2529.5	0.60	0.182
4	+	+		+		+	+	+		33	-2527.9	2.22	0.081
5	+			+	+	+	+	+	+	32	-2527.0	3.15	0.051
6	+	+			+	+	+	+	+	31	-2526.3	3.89	0.035
7	+	+		+	+	+	+	+	+	32	-2526.2	3.98	0.033
8	+				+	+	+	+	+	31	-2525.7	4.40	0.027
9	+	+		+		+	+	+	+	35	-2525.0	5.14	0.019
10	+			+	+		+	+	+	31	-2524.0	6.18	0.011
**Detritivore-mediated decomposition**													
1	+	+				+		+		30	-2470.1	0.00	0.158
2	+	+		+		+		+		31	-2469.2	0.93	0.100
3		+				+	+	+		32	-2468.8	1.32	0.082
4	+					+	+	+		33	-2468.4	1.77	0.065
5	+	+	+			+		+		31	-2468.1	2.01	0.058
6						+	+	+		33	-2468.0	2.15	0.054
7					+	+	+	+		33	-2467.3	2.87	0.038
8			+			+	+	+		34	-2467.0	3.15	0.033
9		+		+		+		+	+	32	-2466.8	3.35	0.030
10		+	+			+	+	+		33	-2466.5	3.61	0.026

Supplementary Table 2. Results of generalized additive models exploring significant interactions between detritivore variables (see Table 1) and latitude as categorial predictor (tropical, ≤ 23º; temperate, 24-60º; polar, > 60º) to total and detritivore-mediated litter decomposition. We show effective degrees of freedom (edf) and values of *F* and *P* for each factor.

Effect	edf	*F*	*P*
**Total decomposition**			
Diversity, tropical	3.08	10.66	<0.001
Diversity, temperate	1.67	6.71	0.001
Diversity, polar	1.00	0.09	0.765
Abundance, tropical	1.00	0.35	0.553
Abundance, temperate	4.00	4.25	<0.001
Abundance, polar	1.00	2.14	0.146
Biomass, tropical	1.00	0.50	0.482
Biomass, temperate	3.27	4.95	<0.001
Biomass, polar	1.00	5.04	0.026
**Detritivore-mediated decomposition**			
Diversity, tropical	2.63	3.96	0.008
Diversity, temperate	3.08	2.46	0.054
Diversity, polar	1.00	0.59	0.444
Abundance, tropical	1.00	1.35	0.247
Abundance, temperate	8.13	21.68	<0.001
Abundance, polar	1.00	4.36	0.038
Biomass, tropical	3.42	4.47	0.515
Biomass, temperate	1.00	22.10	0.002
Biomass, polar	1.00	22.10	<0.001

Supplementary Table 3. Families of detritivores with predominantly Laurasian, Gondwanan or global distribution, and total number of individuals (N) found in this study. Note that some families (Baetidae, Chironomidae and Leptophlebiidae) have few litter-feeding detritivores; here we refer to the distribution of litter-feeding detritivore taxa within these families, rather than to whole families.

**Laurasian distribution**		**Gondwanan distribution**		**Global distribution**	
Family	N	Family	N	Family	N
Nemouridae	1167	Calamoceratidae	412	Lepidostomatidae	2101
Gammaridae	897	Janiridae	139	Leptophlebiidae	880
Leuctridae	665	Atyidae	77	Leptoceridae	358
Asellidae	326	Elmidae	32	Chironomidae	232
Capniidae	290	Perthiidae	26	Tipulidae	133
Sericostomatidae	207	Crambidae	20	Baetidae	45
Taeniopterygidae	206	Palaemonidae	10		
Calocidae	180	Potamonautidae	8		
Psychodidae	149	Odontoceridae	6		
Limnephilidae	141	Hyalellidae	4		
Eusiridae	114	Helicophidae	2		
Chloroperlidae	106	Pisuliidae	2		
Ephemerellidae	93	Blaberidae	1		
Gripopterygidae	78	Ptilodactylidae	1		
Crangonyctidae	39				
Ephemeridae	35				
Austroperlidae	26				
Scirtidae	18				
Diamphipnoidae	16				
Limoniidae	16				
Paramilitidae	11				
Phryganeidae	10				
Notonemouridae	6				
Brachycentridae	4				
Pteronarcyidae	3				
Tanyderidae	2				

Supplementary Table 4. Location of study sites [country, region, stream name, biome, decimal latitude (Lat) and longitude (Long) and altitude (degrees) and altitude (Alt; m asl)]; main climatic variables [mean annual temperature (MT, °C); annual precipitation (AP, mm); temperature seasonality (TS, standard deviation of monthly mean values x 100) and precipitation seasonality (PS, coefficient of variation of monthly mean values)]; and main physicochemical characteristics [stream average width (Wi; m); pH; nitrate-N (NO3; µg L^{-1}) and phosphate–P (PO4; µg L^{-1})]. Sites are ordered by increasing absolute latitude. NP, National Park; NT, Northern Territory; QLD, Queensland; GA, Georgia; WA, Western Australia; IL, Illiniois; MD, Maryland; NY, New York; MI, Michigan; MO, Montana BC, British Columbia. Biomes: TrS; tropical wet forest, TrWF; xeric shrubland, XeS (also termed desert, although aridity was low in riparian forests in our study regions); Mediterranean forest, MeF; temperate broadleaf forest, TeBF; temperate coniferous forest, TeCF; and tundra, Tu.

Country	Region	Stream name	Biome	Lat	Long	Alt	MT	AP	TS	PS	Wi	pH	NO3	PO4
Kenya	Nakuru	Njoro	TrS	-0.37	35.93	2263	15.8	954	72	43	4.4	8.1	2485.0	6.9
Kenya	Narok	Ngetunyek	TrWF	-0.71	35.46	2096	16.5	1043	74	48	2.3	7.4	319.0	11.5
Brazil	Amazonas	Acará 23	TrWF	-2.95	-59.96	58	27.2	2188	48	439	2.6	4.6	15.7	26.3
Malaysia	Kuala Lumpur	Ampang	TrWF	3.17	101.78	176	26.5	2502	37	27	1.6	7.4	600.0	5.0
Brazil	Rio Grande do Norte	Pitimbú	TrWF	-5.92	-35.18	50	25.8	1402	108	71	4.2	6.5	1050.0	1.4
Panama	Chiriquí	Caldera	TrWF	8.85	-82.50	1825	15.9	2266	58	55	3.7	6.9	666.0	16.0
Guinea	Faranah	Djigbè	TrWF	9.16	-10.56	549	24.2	2131	139	76	3.8	7.5	140.0	0.7
India	Tamil Nadu	Thadaganachiamman	TrWF	10.09	77.25	740	19.3	2150	130	76	3.2	6.9	980.0	32.0
Venezuela	Yaracuy	Herrera 1	XeS	10.29	-68.65	123	24.2	1253	45	55	4.3	8.3	1439.5	1.1
Brazil	Bahia	Morro Fervido	XeS	-12.99	-41.34	950	20.3	940	130	63	0.8	3.9	9.1	54.8
Australia	Litchfield NP (NT)	Shady	TrS	-13.10	130.78	111	26.9	1422	210	109	2.2	8.0	7.0	0.7
Guatemala	Laguna Lauchá NP	Machacas	TrWF	15.95	-90.68	194	25.7	3147	148	62	3.6	6.5	53.8	2.7
Puerto Rico	El Yunque NP	Prieta	TrWF	18.31	-65.75	350	22.1	2961	131	27	2.1	7.2	55.2	3.1
Australia	Paluma Range NP (QLD)	Birthday	TrWF	-18.98	146.16	820	20.2	2584	344	67	8.6	6.3	19.0	2.5
Brazil	Minas Gerais	RCA52	TrS	-19.16	-47.01	891	21.1	1545	169	84	4.9	6.6	12.1	0.7
Australia	Tamborine NP (QLD)	Cedar	TeBF	-27.90	153.18	240	18.6	1325	373	41	5.7	6.9	706.0	3.0
United States	Toombs (GA)	15 Mile	TeCF	32.45	-82.06	58	18.6	1198	685	23	7.9	7.2	96.8	140.8
South Africa	Western Cape	Lourens	Me	-34.08	18.89	85	16.1	852	358	63	6.3	7.1	585.0	5.0
Australia	South West (WA)	Warren	Me	-34.51	116.10	93	14.9	1131	332	69	8.7	7.8	6.3	0.7
Japan	Honshu	Komori	TeBF	35.83	138.53	1080	7.6	1653	880	53	6.0	7.5	585.0	7.2
Chile	Biobío	Nonguén	TeBF	-36.88	-72.99	95	11.2	1502	304	84	5.0	7.4	32.8	20.0
Spain	Sierra Nevada	Alhama	Me	37.20	-3.25	1374	9.3	758	688	44	2.5	7.5	89.5	3.5
United States	Balcom (IL)	Big	TeBF	37.42	-89.17	123	13.6	1196	932	16	8.8	7.7	820.0	76.0
Australia	Victoria	Keppel	TeBF	-37.45	145.76	415	10.8	1592	465	39	1.9	7.5	52.5	5.0
Portugal	Lousã (Coímbra)	Cerdeira	Me	40.09	-8.20	531	12.5	1288	504	58	2.1	7.2	60.5	7.5
Argentina	Patagonia Andina	Rojizo	TeBF	-41.23	-71.30	1120	5.9	810	443	62	1.2	7.9	236.2	11.5
United States	Ithaca (NY)	Cascadilla	TeBF	42.43	-76.45	275	7.7	930	939	20	4.3	8.0	250.8	6.8
United States	Macomb (MI)	Stoney	TeBF	42.79	-83.09	258	8.7	792	1009	23	4.6	6.6	207.1	71.7
Australia	Tasmania	Browns	TeBF	-42.92	147.25	430	10.5	969	318	18	3.1	7.4	3.3	6.0

Country	Region	Stream name	Biome	Lat	Long	Alt	MT	AP	TS	PS	Wi	pH	NO3	PO4
Japan	Hokkaido	Toyohira	TeCF	42.93	141.16	450	5.4	1359	1009	23	3.7	7.1	919.4	0.7
Spain	Cordillera Cantábrica	Agüera	TeBF	43.21	-3.27	305	12.5	1081	436	28	2.3	7.2	675.0	1.7
France	Occitanie	Peyreblanque	TeBF	43.42	2.22	752	10.1	908	588	14	2.3	6.6	630.1	1.3
United States	Flathead lake NP (MT)	Roy's	TeCF	47.88	-114.03	897	6.0	610	800	22	3.6	7.9	550.9	9.0
Canada	Vancouver Coast (BC)	East	TeCF	49.27	-122.57	165	8.8	1717	573	52	2.0	6.6	675.5	2.2
Poland	Malopolska	Krzyworzeka	TeCF	49.86	20.12	266	7.9	728	799	47	7.9	8.3	1516.5	15.1
Germany	NS Ruppiner Land	Kunster	TeBF	53.02	12.75	62	8.4	575	688	24	2.7	7.5	240.1	17.5
Sweden	Uppsala	Lafssjon	TeBF	60.03	17.81	61	5.6	564	790	32	4.4	6.9	427.0	1.0
Finland	Finnish Lapland	Garnjargajohka	Tu	69.93	27.14	75	-0.8	425	824	43	2.8	7.1	3.3	0.7

Supplementary Table 5. Plant species used in the different litter mixtures and place of collection (country and decimal latitude and longitude in degrees).

Mixture	Species	Country	Lat	Long
I	*Alnus acuminata*	Ecuador	-0.67	-77.92
	Alnus glutinosa	Spain	43.21	-3.27
	Alnus incana	Sweden	60.03	17.82
II	*Ficus insipida*	Costa Rica	10.43	-84.01
	Ficus natalensis	Kenya	-0.37	35.92
	Ficus dulciaria	Ecuador	-0.59	-77.88
III	*Fagus sylvatica*	France	43.42	2.19
	Quercus prinus	USA	39.22	-76.70
	Castanea sativa	Portugal	41.18	-8.35
IV	*A. acuminata*			
	F. insipida			
	F. sylvatica			
V	*A. glutinosa*			
	F. natalensis			
	Q. prinus			
VI	*A. incana*			
	F. dulciaria			
	C. sativa			

Supplementary Figures

Supplementary Figure 1. Box plot showing the median, interquartile range and minimum-maximum range of total decomposition (prop. litter mass loss) at the end of the experiment in each biome; n = 38 independent experiments. Tu, tundra; TeBF, temperate broadleaf forest; TeCF, temperate coniferous forest; MeF, Mediterranean forest; XeS, xeric shrubland; TrWF, tropical wet forest; and TrS, tropical savanna.

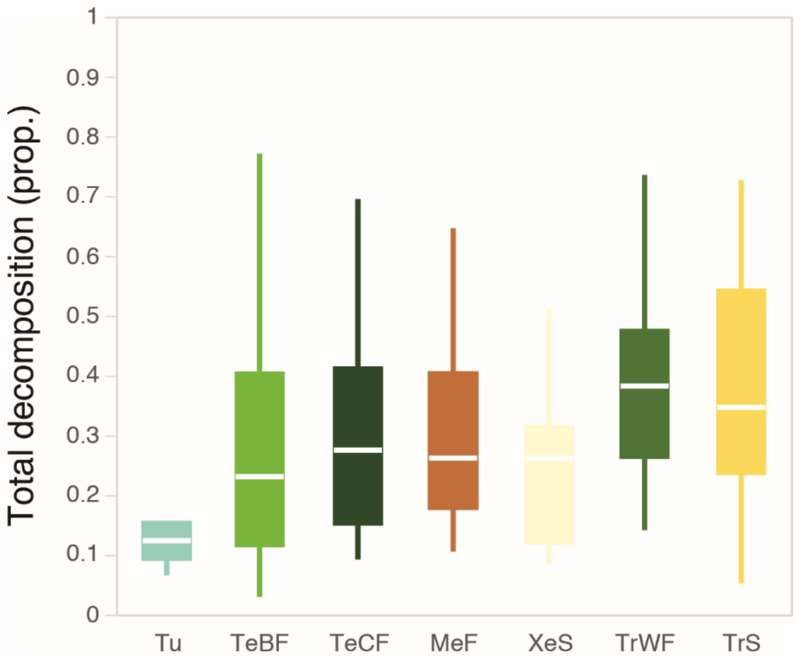

Supplementary Notes

Supplementary Note 1. Funding sources used to conduct research in different regions. **Argentina**: ANCYPT (PICT.2016-959). **Australia, Tasmania**: Australian Research Council Discovery Program (ARC- DP) DP190102837). **Brazil, Bahia**: Coordenação de Aperfeiçoamento de Pessoal de Nível Superior (CAPES ref. 88882.347849/2019-01); Conselho Nacional de Desenvolvimento Científico e Tecnológico (CNPq ref. 424661/2016-0). **Brazil, Manaus**: Programa de Apoio à Fixação de Doutores no Amazonas FIXAM/AM (Amazonas State Research Foundation, FAPEAM); Conselho Nacional de Desenvolvimento Científico e Tecnológico (CNPq; ref. 308970/2019-5); INCT ADAPTA II – CNPq (ref. 465540/2014-7); FAPEAM (ref. 062.1187/2017); CAPES–Coordination for the Improvement of Higher Education Personnel. **Brazil, Minas Gerais**: Programa Peixe-Vivo of Companhia Energética de Minas Gerais (CEMIG) and P&D Aneel-Cemig GT-599 and GT-611; Conselho Nacional de Desenvolvimento Científico e Tecnológico (303380/2015-2); Coordenação de Aperfeiçoamento de Pessoal de Nível Superior (CAPES) Finance Code 001. **Brazil, Rio Grande do Sul**: CNPq grant 305203/2017-7 and 421288/2017-5. **Chile**: ANID/FONDAP/15130015. **Canada**: Natural Sciences and Engineering Research Council of Canada. **Costa Rica** (litter collection and export): US National Science Foundation, grant DEB-1938843. **Finland**: Academy of Finland (grant no. 318230). **Guatemala**: Education for Nature Program from the World Wild Fund and LASPAU-Fulbright Program. **India**: Science and Engineering Research Board, New Delhi (ref. ECR/2016/000191/LS). **Japan, Sapporo**: Ministry of Land, Infrastructure, Transport, and Tourism of Japan; JSPS Grant-in-Aid for Scientific Research (B) (18H03407). **Kenya, Eldoret**: International Foundation for Science (Research Grant No. A/5810-1). **Panama**: National Secretariat for Science, Technology and Innovation (SENACYT; ref. APY-GC-2018B-052 contract no. 259-2018 and Scholarship contract no. 001-2015-AC); National Research System of Panama (SNI; contract no. 186-2018-AC); Scholarship IFARHU-SENACYT (contract no. 270-2018-1011-GG). **Portugal**: IATV and Portuguese Foundation for Science and Technology (FCT; strategic project UIDP/04292/2020 granted to MARE). **Spain, Almería**: 2014-2020 Operational Programme FEDER Andalusia, Spain (ref. UAL18-RNM-B006-B). **Spain, Basque Country**: Basque Government funds (ref. IT951-16); Spanish Ministry for Science, Innovation and Universities (ref. RTI2018-095023- B-I00). **Other regions:** funding was obtained from host institutions.
