# Supplementary material for: Impacts of detritivore diversity loss on instream decomposition are greatest in the tropics
Source: Nat Commun. 2021 Jun 17;12:3700. doi: 10.1038/s41467-021-23930-2 (PMC8211652; doi:10.1038/s41467-021-23930-2)
Supplement: Supplementary file 3 — Reporting Summary [file 41467_2021_23930_MOESM3_ESM.pdf]

nature research

Corresponding author(s): Luz Boyero

Last updated by author(s): May 6, 2021

Reporting Summary

Nature Research wishes to improve the reproducibility of the work that we publish. This form provides structure for consistency and transparency in reporting. For further information on Nature Research policies, see our Editorial Policies and the Editorial Policy Checklist.

Statistics

For all statistical analyses, confirm that the following items are present in the figure legend, table legend, main text, or Methods section.

n/a	Confirmed	
☐	☒	The exact sample size (n) for each experimental group/condition, given as a discrete number and unit of measurement
☐	☒	A statement on whether measurements were taken from distinct samples or whether the same sample was measured repeatedly
☐	☒	The statistical test(s) used AND whether they are one- or two-sided<br>*Only common tests should be described solely by name; describe more complex techniques in the Methods section.*
☐	☒	A description of all covariates tested
☐	☒	A description of any assumptions or corrections, such as tests of normality and adjustment for multiple comparisons
☐	☒	A full description of the statistical parameters including central tendency (e.g. means) or other basic estimates (e.g. regression coefficient) AND variation (e.g. standard deviation) or associated estimates of uncertainty (e.g. confidence intervals)
☐	☒	For null hypothesis testing, the test statistic (e.g. F, t, r) with confidence intervals, effect sizes, degrees of freedom and P value noted<br>*Give P values as exact values whenever suitable.*
☒	☐	For Bayesian analysis, information on the choice of priors and Markov chain Monte Carlo settings
☐	☒	For hierarchical and complex designs, identification of the appropriate level for tests and full reporting of outcomes
☒	☐	Estimates of effect sizes (e.g. Cohen's d, Pearson's r), indicating how they were calculated

Our web collection on statistics for biologists contains articles on many of the points above.

Software and code

Policy information about availability of computer code

Data collection	No software was used for data collection.
Data analysis	All data analyses were performe in R statistical software (v. 4.0.2) packages mgvc (v. 1.8.31), MuMIn (v. 1.43.17), nlme (v. 3.1.143), multcomp (v. 1.4.13) and vegan (v. 2.5.6).

For manuscripts utilizing custom algorithms or software that are central to the research but not yet described in published literature, software must be made available to editors and reviewers. We strongly encourage code deposition in a community repository (e.g. GitHub). See the Nature Research guidelines for submitting code & software for further information.

Data

Policy information about availability of data

All manuscripts must include a data availability statement. This statement should provide the following information, where applicable:

- Accession codes, unique identifiers, or web links for publicly available datasets
- A list of figures that have associated raw data
- A description of any restrictions on data availability

Data supporting the findings of this study are available at https://figshare.com/s/64c1878e54b92a352072.

Field-specific reporting

Ecological, evolutionary & environmental sciences study design

All studies must disclose on these points even when the disclosure is negative.

Study description	A litter decomposition experiment was conducted in 38 streams, each located in a different region. Regions were distributed worldwide, across a 113º latitudinal gradient. At each site we incubated 30 litterbags coarse-mesh and 30 fine-mesh litterbags equally distributed among 6 types of litter mixture. All litterbags were collected at a single time (23-46 d depending on water temperature).
Research sample	Litterbags are the most common sample units used in decomposition experiments. Our litterbags contained 3 g of leaf litter belonging to 3 species, with different combinations in each litter mixture. Samples collected at the end of the experiment consisted of the remaining leaf litter (which was used to estimate decomposition) and the associated invertebrates (which were used to characterize the litter-consuming detritivore assemblages).
Sampling strategy	Litterbags were collected using a hand net that was placed immediately downstream to avoid the loss of leaf material and invertebrates. Litterbags were then placed in individual ziplock bags and transported to the labortory within coolers. We used 5 litterbags per mixture type and mesh size, with 60 litterbags per site and 2280 in total. The number of litterbags (n = 5) is considered appropriate for litter decomposition studies in streams (4-6 litterbags recommended by Barlöcher 2020 Leaf Mass Loss Estimated by the Litter Bag Technique; in Barlöcher et al, Methods to Study LitterDecomposition; Springer). Replicates in our experiment were streams (n = 38).
Data collection	In this globally-distributed, collaborative study, each contributing team (composed of 2 coauthors) collected samples at their corresponding site. Litterbags were collected as described above and processed in the laboratory. Decomposition was calculated as leaf litter mass loss, based on initial and final leaf litter dry mass. Invertebrates in each sample were counted and identified to calculate abundance and taxon richness. Invertebrate biomass and body size were derived from known equations from the literature.
Timing and spatial scale	The study was conducted from late 2017 to early 2019. Timing differed across sites due to differences in climatic conditions: it was conducted during the dry season at tropical sites and in the autumn at higher latitude sites. At each site, the experiment duration was 23-46 d, with experimental time being determined by water temperature in order to achieve a similar stage of decomposition across sites. The spatial scale of the study was global; at each site, the experiment was conducted in one stream.
Data exclusions	We excluded 5 sites from our original design because 3 were unable to retrieve the litterbags due to extreme climatic events and 2 were not able to provide invertebrate data. We did not exclude any data from our final dataset.
Reproducibility	Our study consists of a experiment that was replicated at 38 sites around the world, which ensures its reproducibility.
Randomization	At each site, litterbags were placed in 5 consecutive stream pools, with one litterbag per litter mixture type and mesh size in each pool.
Blinding	Blinding was not relevant in our study. Litterbags were all deployed and collected from streams and processed identically.

Did the study involve field work? ☒ Yes ☐ No

Field work, collection and transport

Field conditions	Due to the global nature of this study, with 38 sites with completely different characteristics, we do not report field conditions here, but they are rather provided in the paper.
Location	Due to the global nature of this study, with 38 sites located all around the world, we do not report specific locations here, but they are rather provided in the paper.
Access & import/export	Due to the high number of countries involved in this study (23), we are unable to provide here the identifiying information fort all the scientific permits obtained, but we state that we compiied with the requests of each country.
Disturbance	Disturbance at the studied streams was minimal: all the material used in the experiments was retrieved, we did not use any chemicals in situ, and only collected 60 invertebrate samples per site.

Reporting for specific materials, systems and methods

We require information from authors about some types of materials, experimental systems and methods used in many studies. Here, indicate whether each material, system or method listed is relevant to your study. If you are not sure if a list item applies to your research, read the appropriate section before selecting a response.

Materials & experimental systems

n/a	Involved in the study	
[x]	[ ]	Antibodies
[x]	[ ]	Eukaryotic cell lines
[x]	[ ]	Palaeontology and archaeology
[ ]	[x]	Animals and other organisms
[x]	[ ]	Human research participants
[x]	[ ]	Clinical data
[x]	[ ]	Dual use research of concern

Methods

n/a	Involved in the study	
[x]	[ ]	ChIP-seq
[x]	[ ]	Flow cytometry
[x]	[ ]	MRI-based neuroimaging

Animals and other organisms

Policy information about studies involving animals; ARRIVE guidelines recommended for reporting animal research

Laboratory animals	Our study did not involve laboratory animals.
Wild animals	We collected stream invertebrates (insect larvae and small crustaceans).that were contained in the litterbags at the end of the experiment. Invertebrates were trasported to the laboratory within the litterbags. In the laboratory, they were hand-picked and introduced in vials containing 70% ethanol for further identification.
Field-collected samples	We did not work with live animals in the laboratory.
Ethics oversight	No permits were required apart from usual field research permits.

Note that full information on the approval of the study protocol must also be provided in the manuscript.
